# Neutrophil‐to‐lymphocyte ratio, hyperglycemia, and outcomes in ischemic stroke patients treated with intravenous thrombolysis

**DOI:** 10.1002/brb3.1741

**Published:** 2020-07-22

**Authors:** Yani Cheng, Anna Ying, Yanyan Lin, Junru Yu, Ji Luo, Yifan Zeng, Yuanshao Lin

**Affiliations:** ^1^ Department of Neurology The First Affiliated Hospital of Wenzhou Medical University Wenzhou China; ^2^ Department of Neurology The Third Affiliated Hospital of Wenzhou Medical University Wenzhou China

**Keywords:** hyperglycemia, inflammation, Neutrophil‐to‐lymphocyte ratio, outcome, stroke, thrombolysis

## Abstract

**Introduction:**

Increased neutrophil‐to‐lymphocyte ratio (NLR) and hyperglycemia on admission are associated with poor outcomes in acute ischemic stroke (AIS) patients. We sought to evaluate the combined effect of increased NLR and hyperglycemia on the prognosis of ischemia stroke treated with intravenous thrombolysis (IVT).

**Methods:**

Patients with acute ischemic stroke receiving IVT treatment were prospectively enrolled. All participants were followed for 3 months. According to the levels of NLR and blood glucose, patients were categorized into four groups: high NLR or nonhigh NLR with or without hyperglycemia. The associations between NLR values with or without hyperglycemia and outcomes of stroke after thrombolysis were assessed by multivariable logistic regression analysis.

**Results:**

Among the 381 stroke patients (median age 68 years, 61.68% man) included, 155 (40.68%) had a poor outcome (modified Rankin Scale score 3–6) during 3 months. After multivariate adjustment, high NLR with hyperglycemia increased the risk of 3‐month poor outcome (OR = 4.42; 95% CI, 2.13–9.16), early neurological deterioration (END) (OR = 4.81; 95% CI, 2.08–11.12), and 3‐month mortality (OR = 6.56; 95% CI, 1.92–22.40). A significant multiplicative interaction of NLR and blood glucose on 3‐month poor outcome in ischemic stroke patients after thrombolysis was observed.

**Conclusions:**

Ischemic stroke patients with concurrent high NLR and hyperglycemia increased risks of END, 3‐month poor outcome, and mortality after thrombolysis.

## Graphical abstract

The present study found that concurrent high NLR and hyperglycemia increased risks of END, 3‐month poor outcome, and mortality after thrombolysis. Moreover, high NLR and hyperglycemia exerted a significant multiplicative interaction on poor outcome at 3 months, and high NLR was merely a strong predictor of 3‐month poor outcome and END in patients with hyperglycemia.

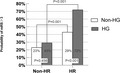

## 1 BACKGROUND

Acute ischemic stroke (AIS) is a grave threat to global healthcare system, which is accompanied by high disability and mortality (Fan, Gui, Chai, & Wei, 2018). Intravenous thrombolysis with recombinant tissue plasminogen activator (rtPA) is considered as the most effective medical reperfusion treatment within 4.5 hr after the onset of symptoms in patients with AIS (Emberson et al., 2014). However, increased risks of side effects following IVT such as cerebral hemorrhage (HT) may partially offset the benefits of reperfusion and even lead to neurological deterioration (Jickling et al., 2014; Seners et al., 2014). Therefore, the detection of useful biomarkers is essential to an early risk assessment and effective therapy after thrombolysis.

Recently, mounting attention has been drawn to the prognostic role of inflammation in AIS. AIS is no longer believed to be only a vascular disease, but also an immune‐mediated condition (Jiang, Moyle, Soule, Rote, & Chopp, 1995). Neutrophil, a critical immune responder to ischemic brain insult, has been suggested to accumulate accompanying progression of infarct lesion and exacerbate brain damage (Ishikawa, Zhang, Nanda, & Granger, 2004; Jiang, Moyle, Soule, Rote, & Chopp, 1995). In addition, neutrophil was associated with the higher disability and mortality rates in stroke patients receiving rtPA treatment (Liu et al., 2019; Malhotra et al., 2018). Similarly, neutrophil‐to‐lymphocyte ratio (NLR) has been widely investigated and has emerged as a novel inflammation marker. Previous studies have demonstrated NLR as a predictor of major disability (Liu et al., 2019), mortality (Tokgoz, Keskin, Kayrak, Seyithanoglu, & Ogmegul, 2014), and HT (Guo et al., 2016). Admission hyperglycemia is prevalent in more than a third of AIS patients (Dungan, Braithwaite, & Preiser, 2009) and plays an important role in prognosis of stroke. Increased risks of poor functional outcomes, mortality, and HT have been observed for high admission blood glucose level in stroke patients treated with IVT (Lin et al., 2018; Poppe et al., 2009). Moreover, several studies have suggested the deleterious effects of hyperglycemia are attributable to the reperfusion following thrombolysis (Alvarez‐Sabín et al., 2004; Putaala et al., 2011). Interestingly, hyperglycemia has been shown to enhance endothelial activation and neutrophil infiltration in stroked rats (Justicia et al., 2017). A clinical study has demonstrated a strong association between NLR and hyperglycemia in patients with spontaneous intracerebral hemorrhage (Zhang et al., 2019). However, the combined relationship between NLR and hyperglycemia in AIS patients after rtPA treatment has not been well studied.

In the present study, we conducted a prospective study to investigate the combined effects of the pattern of high NLR and hyperglycemia on poor outcomes in ischemic stroke patients after thrombolysis.

## 2 METHODS

### 2.1 Study population

Consecutive AIS patients at the First Affiliated Hospital of Wenzhou Medical University from January 2016 to January 2019 were prospectively enrolled in our study. The inclusion criteria for enrollment were (a) older than 18 years, (b) diagnosis of AIS and treatment with IV rtPA within 4.5 hr of symptom onset. Exclusion criteria included (a) evidence of acute infection at admission or any infection that occurred during the first 48h after admission; (b) cancer, chronic inflammation disease, autoimmune disease, or immunosuppressive drug use; and (c) patients who received a bridging therapy consisting of IV rtPA followed by endovascular therapy. Finally, a total of 381 ischemic stroke patients were included in the current study. The present study was approved by the Ethics Committee of the First Affiliated Hospital of Wenzhou Medical University, and all patients or their relatives gave informed consent.

### 2.2 Baseline assessment

Baseline information for all patients including demographics, risk factors (blood pressure, smoking, and alcohol drinking status), onset‐to‐treatment time, and medical history (diabetes, hypertension, dyslipidemia, atrial fibrillation, and history of stroke) were recorded on admission. Stroke severity was assessed by trained neurologists with the National Institutes of Health Stroke Scale (NIHSS) on admission. Venous blood samples were collected within 24 hr of hospital admission after overnight fasting. A complete blood count was determined using the automated hematology analyzer (XE‐2100, Sysmex Company, Japan) within 60 min after blood sample collection. NLR was calculated as the neutrophil count divided by the lymphocyte count. Hyperglycemia was defined as a fasting glucose level of ≥110 mg/dl (6.1 mmol/L). Diabetes mellitus was defined as HbA1c ≥ 6.5% or current use of oral or parenteral antidiabetic medicines. Hypertension was defined as a systolic blood pressure ≥140 mm Hg or a diastolic pressure ≥90 mm Hg persisting after the acute stage of ischemic stroke, or by antihypertensive medication use prior to admission. Dyslipidemia was defined as a fasting plasma cholesterol level ≥220 mg/dl, a fasting plasma triglyceride level ≥150 mg/dl, or by the use of lipid‐lowering medications prior to admission.

### 2.3 Outcome measures

The primary outcome was poor functional outcome (modified Rankin Scale (mRS) score, 3–6) at 3 months after stroke. Secondary outcomes were early neurological deterioration (END) and 3‐month mortality. END was defined as an increment of at least 4 point within 24 hr following IVT compared to the NIHSS scores on admission (Seners et al., 2014). All patients were followed up prospectively by telephone interview at 3 months conducted by a trained neurologist.

### 2.4 Statistical analysis

The results were presented as the mean (standard deviation, *SD*) or median (interquartile range, IQR) for continuous variables and percentages for categorical variables. NLR at baseline was categorized into two levels according to NLR median value in all participants (High NLR ≥ 4.0; Nonhigh NLR < 4.0). The study patients were then classified into four groups in accordance with their NLR and fasting glucose levels: nonhigh NLR and nonhyperglycemia (Non‐HR and non‐HG), nonhigh NLR and hyperglycemia (Non‐HR and HG), high NLR and nonhyperglycemia (HR and non‐HG), and high NLR and hyperglycemia (HR and HG). The differences among four groups were tested by one‐way analysis of variance (ANOVA) or the Kruskal–Wallis *H* test for continuous variables, as appropriate. Comparisons of two groups in terms of continuous variables were evaluated with Student's *t* test and Mann–Whitney *U* test as appropriate. Categorical variables were compared using the chi‐square test. Logistic regression analysis was performed to estimate the probability of having poor outcomes of stroke after IVT and 95% CI for each risk factor category stratified by NLR and glucose levels, adjusting for age, gender, and other conventional confounders. We tested the statistical significance of NLR category × glucose status in multivariable logistic model to examine the multiplicative interaction of elevated NLR and hyperglycemia.

## 3 RESULTS

In this study, a total of 533 AIS patients treated with rtPA were screened, and 152 patients were excluded due to occurrence of acute infection (*n* = 68), bridging therapy (*n* = 27), a history of cancer (*n* = 8), autoimmune disease or immunosuppressive drug use (*n* = 14), and losing to follow up (*n* = 35).

Of the 381 patients in the study sample (median age 68 years, 61.68% man), 155 (40.7%) had poor outcome at 3 months after IVT. Table 1 shows the baseline characteristics of the study population based on the development of good and poor outcomes. Patients with poor outcome were more likely to be older, had higher NLR and blood glucose values and baseline NIHSS scores, and more of them suffered from hypertension and atrial fibrillation compared to the good group. However, there was no significant difference in gender, HbA1c, and the incidence of diabetes.

**TABLE 1:** The baseline characteristics of good and poor functional outcomes at 3 months

Characteristics	Total (*n* = 381)	Good outcome (*n* = 226)	Poor outcome (*n* = 155)	*p* Value
Age, years, median (IQR)	68 (59–76)	65 (25–72)	73 (64–81)	<.001
Gender, male, *n* (%)	235 (61.68)	136 (60.18)	99 (63.87)	.466
Current cigarette smoking, *n* (%)	147 (38.58)	98 (43.36)	49 (31.61)	.021
Current alcohol drinking, *n* (%)	108 (28.35)	71 (31.42)	37 (23.87)	.108
Hypertension, *n* (%)	286 (75.07)	155 (68.58)	131 (84.52)	<.001
Diabetes, *n* (%)	91 (23.89)	52 (23.01)	39 (25.16)	.628
Hyperlipidemia, *n* (%)	142 (37.27)	96 (42.48)	46 (29.68)	.011
Previous stroke, *n* (%)	42 (11)	18 (7.96)	24 (15.48)	.021
Atrial fibrillation, *n* (%)	105 (27.56)	41 (17.90)	64 (41.29)	<.001
Onset‐to‐treatment time, min, median (IQR)	200 (149.50–241)	200 (150–240)	200 (160–253)	.547
Baseline NIHSS score, median (IQR)	7 (3.50–11)	5 (3–8)	11 (6–14)	<.001
SBP, mm Hg, mean ± *SD*	154.61 ± 23.37	152.69 ± 22.67	157.43 ± 24.15	.052
DBP, mm Hg, mean ± *SD*	85.39 ± 15.74	85.38 ± 15.56	85.40 ± 16.06	.995
NLR, *n* (%)	3.94 (2.58–6.71)	3.29 (2.27–5.05)	5.72 (3.41–9.05)	<.001
NLR ≥ 4, *n* (%)	186 (48.82)	64 (28.31)	82 (52.90)	<.001
Blood glucose, mmol/L, mean ± *SD*	5.50 (4.80–6.80)	5.10 (4.61–6.37)	6.10 (5.10–7.40)	<.001
Blood glucose ≥ 6.1, *n* (%)	146 (38.32)	79 (34.96)	107 (69.03)	<.001
TG, mmol/L, median (IQR)	1.23 (0.89–1.76)	4.89 (4.07–5.71)	4.81 (4.08–5.62)	<.001
TC, mmol/L, median (IQR)	4.87 (4.08–5.68)	1.36 (0.99–1.88)	1.09 (0.79–1.38)	.907
LDL‐C, mmol/L, mean ± *SD*	2.89 ± 0.95	2.86 ± 0.91	2.94 ± 1.00	.452
HDL‐C, mmol/L, median (IQR)	1.10 (0.92–1.33)	1.06 (0.92–1.26)	2.85 (2.19–3.37)	.448
HbA1c (%), median (IQR)	5.8 (5.5–6.5)	5.8 (5.5–6.5)	5.9 (5.5–6.6)	.426
TOAST classification				
Large‐artery atherosclerosis, *n* (%)	148 (38.85)	81 (35.84)	67 (43.23)	<.001
Cardioembolic, *n* (%)	109 (28.61)	50 (22.12)	59 (38.06)	
Small‐artery occlusion, *n* (%)	78 (20.47)	74 (32.74)	4 (2.58)	
Other etiology, *n* (%)	2 (0.52)	2 (0.88)	0 (0)	
Undetermined etiology, *n* (%)	44 (11.55)	19 (8,4)	25 (16.13)	

Abbreviations: DBP, diastolic blood pressure; HbA1c, Hemoglobin A1c; HDL, high‐density lipoprotein cholesterol; IQR, interquartile range; LDL‐C, low‐density lipoprotein cholesterol; NIHSS, National Institutes of Health Stroke Scale; NLR, neutrophil‐to‐lymphocyte ratio; SBP, systolic blood pressure; *SD*, standard deviation; TC, total cholesterol; TG, triglyceride.

**FIGURE 1:**
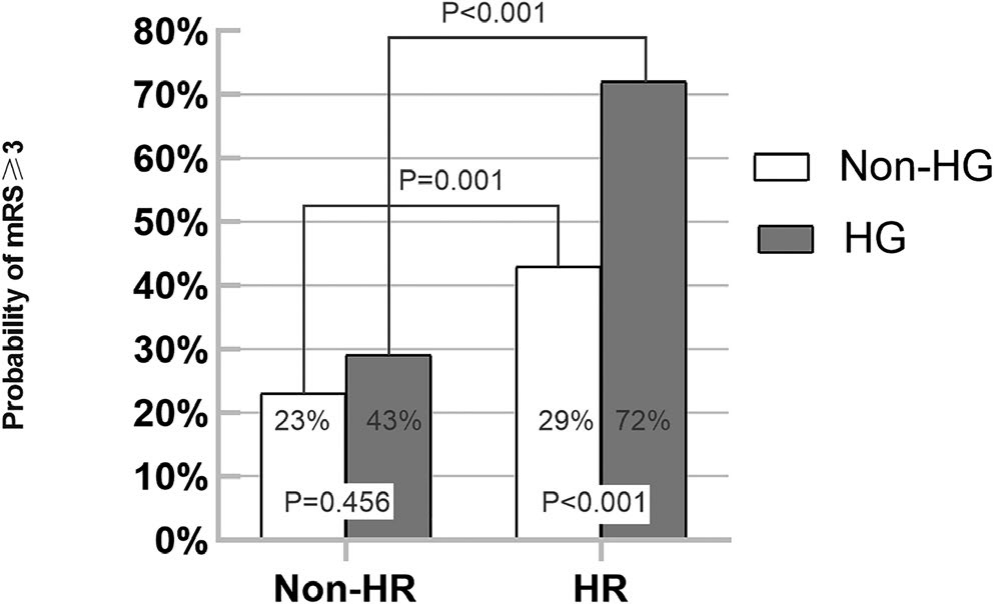
Comparison of outcomes between NLR values with or without hyperglycemia. HG, hyperglycemia; HR, high neutrophil‐to‐lymphocyte ratio; mRS, modified Rankin Scale

**FIGURE 2:**
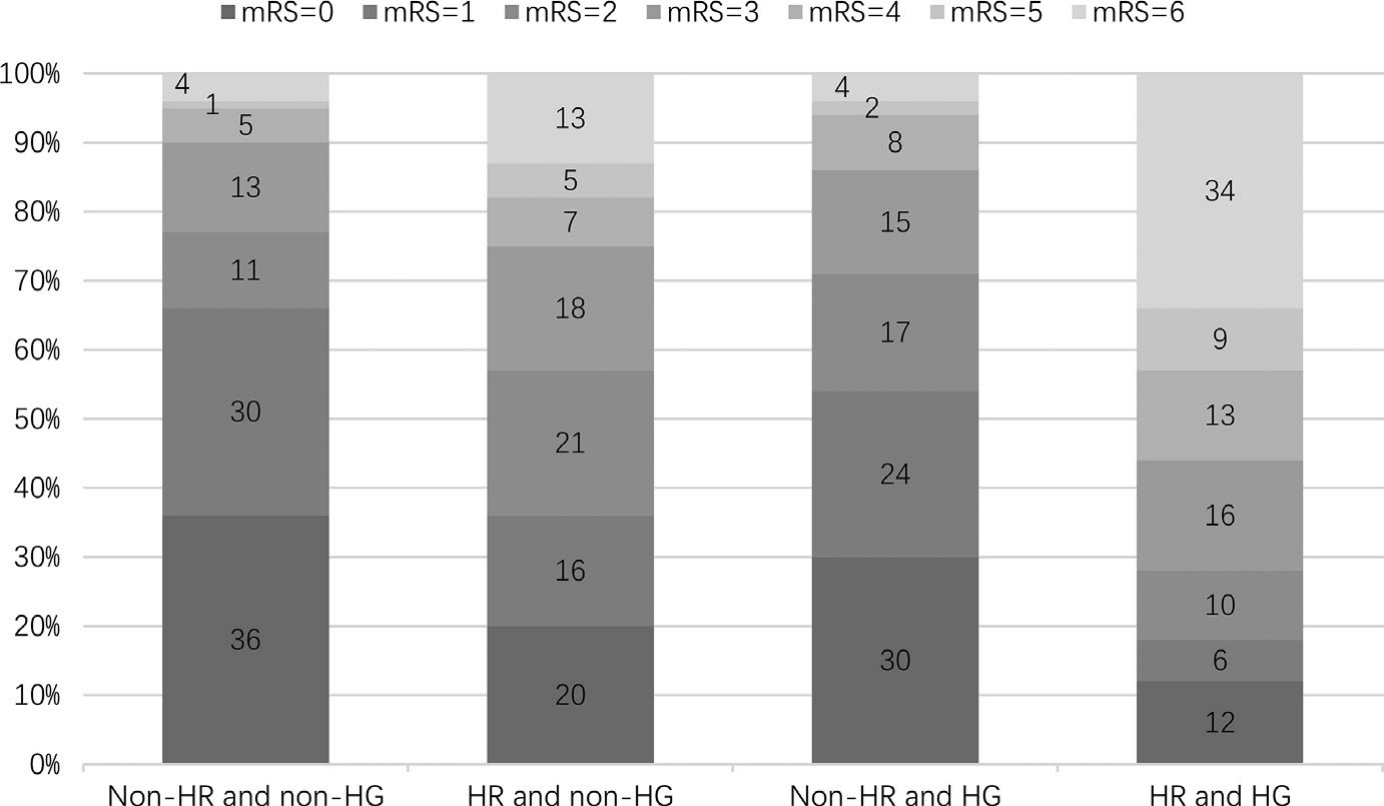
Distribution of functional outcomes at 3 months, according to the different NLR levels in patients with or without hyperglycemia. HG, hyperglycemia; HR, high neutrophil‐to‐lymphocyte ratio; mRS, modified Rankin Scale

When 3‐month clinical outcomes were compared within each NLR levels, in patients with high NLR levels, there was a trend toward worse outcome with hyperglycemia (mRS score ≥ 3; 29% vs. 72%, *p* < .001), but no significant difference of outcomes was discovered in patients with low NLR levels (*p* = .456). For the comparison of different NLR levels, high NLR levels were more likely to have poor functional outcome in stroke patients with or without hyperglycemia (all *p* < .001; Figure 1). When patients were categorized into four groups according to the levels of NLR and blood glucose, the subjects with both high NLR and hyperglycemia accounted for 24.4% of the total studied population. These subjects were more likely to be elderly and have vascular risk factors including hypertension, hyperlipidemia, and diabetes (Table 2). In patients with high NLR and hyperglycemia, there is an increase in the scores of 4, 5, and 6, but a decrease in the scores of 0, 1, and 2 (Figure 2).

**TABLE 2:** The baseline characteristics of NLR with hyperglycemia or not in stroke patients after IVT

Characteristics	Non‐HR and non‐HG (*n* = 142)	HR and non‐HG (*n* = 93)	Non‐HR and HG (*n* = 53)	HR and HG (*n* = 93)	*p* Value
Age, years, median (IQR)	65 (56–74)	72 (60–78.5)	67 (59–73)	69 (61.25–79)	.003
Gender, male, *n* (%)	85 (59.86)	59 (63.44)	36 (67.92)	55 (59.14)	.694
Current cigarette smoking, *n* (%)	68 (47.89)	33 (35.48)	20 (37.74)	26 (2.80)	.018
Current alcohol drinking, *n* (%)	47 (33.10)	28 (30.11)	15 (16.13)	18 (19.35)	.143
Hypertension, *n* (%)	94 (66.20)	73 (78.49)	44 (47.31)	75 (80.65)	.02
Diabetes, *n* (%)	12 (8.45)	3 (3.22)	35 (37.63)	41 (44.09)	<.001
Hyperlipidemia, *n* (%)	51 (35.92)	27 (29.03)	31 (33.33)	33 (35.48)	.004
Previous stroke, *n* (%)	13 (9.15)	8 (8.60)	7 (7.50)	14 (15.05)	.414
Atrial fibrillation, *n* (%)	29 (20.42)	28 (30.11)	16 (17.20)	32 (34.41)	.098
Onset‐to‐treatment time, min, median (IQR)	203 (165–244)	190 (150– 240)	228 (176– 259)	200 (149– 240)	.261
Baseline NIHSS score, median (IQR)	5 (3–7)	8 (5–13.5)	5 (3–8)	11 (7–14)	<.001
SBP, mm Hg, mean ± *SD*	152.05 ± 19.74	152.77 ± 23.59	157.40 ± 23.59	158.77 ± 28.24	.111
DBP, mm Hg, mean ± *SD*	83.80 ± 13.30	82.03 ± 18.01	90.58 ± 15.15	88.20 ± 16.02	.002
NLR, median (IQR)	2.59 (2.13–3.21)	6.09 (4.72–8.65)	2.72 (1.84–3.20)	7.93 (5.72–10.35)	<.001
Blood glucose, mmol/L, median (IQR)	4.80 (4.40–5.20)	5.10 (4.65–5.55)	7.10 (6.40–8.30)	7.55 (6.60–10.68)	<.001
TG, mmol/L, median (IQR)	1.32 (1.02–1.74)	1.07 (0.75–1.48)	1.74 (1.11–2.41)	1.16 (0.82–1.59)	<.001
TC, mmol/L, median (IQR)	4.88 (4.23–5.58)	4.68 (3.66–5.48)	4.95 (4.12–5.81)	4.93 (4.17–5.90)	.133
LDL‐C, mmol/L, mean ± *SD*	2.90 ± 0.88	2.72 ± 1.03	2.90 ± 0.78	3.06 ± 1.03	.112
HDL‐C, mmol/L, median (IQR)	1.06 (0.90–1.27)	1.12 (0.92–1.32)	1.03 (0.87–1.19)	1.21 (0.97–1.40)	.009
HbA1c (%), median (IQR)	5.7 (5.4–6)	5.7 (5.5–5.9)	7.2 (6.3–8.35)	6.5 (5.7–8.4)	<.001
TOAST classification	142	93	53	93	.001
Large‐artery atherosclerosis, *n* (%)	48	38	23	39	
Cardioembolic, *n* (%)	30	27	16	36	
Small‐artery occlusion, *n* (%)	48	16	9	5	
Other etiology, *n* (%)	0	1	0	1	
Undetermined etiology, *n* (%)	16	11	5	12	

Abbreviations: DBP, diastolic blood pressure; HbA1c, Hemoglobin A1c; HDL, high‐density lipoprotein cholesterol; HG, hyperglycemia; HR, high neutrophil‐to‐lymphocyte ratio; IQR, interquartile range; LDL‐C, low‐density lipoprotein cholesterol; NIHSS, National Institutes of Health Stroke Scale; NLR, neutrophil‐to‐lymphocyte ratio; SBP, systolic blood pressure; *SD*, standard deviation; TC, total cholesterol; TG, triglyceride.

**FIGURE 3:**
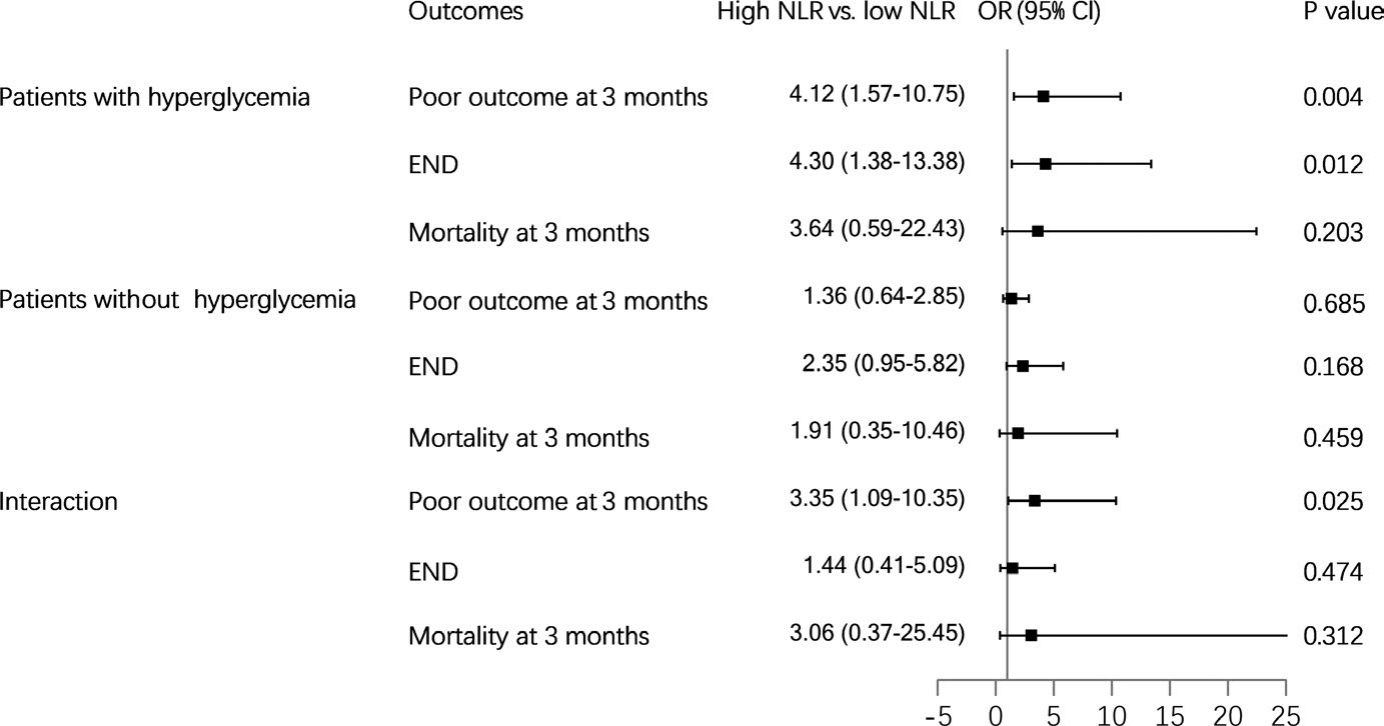
Adjusted ORs ratios of NLR levels for outcomes among ischemic stroke patients with or without hyperglycemia. Adjusted for age, sex, cigarette smoking, alcohol consumption, systolic blood pressure, onset‐to‐treatment time, hypertension, hyperlipidemia, atrial fibrillation, previous stroke, baseline NIHSS score, triglyceride, total cholesterol, low‐density lipoprotein cholesterol, and high‐density lipoprotein cholesterol. CI, confidence interval; OR, odds ratio

The association of NLR and blood glucose with clinical outcomes after thrombolysis was presented in Table 3. In the univariate analysis, patients with both high NLR and hyperglycemia increased the risks of END, 3‐month poor outcome, and mortality (all *p* < .05), compared to those with low NLR and blood glucose. This effect was consistent after adjusting for age, sex, and other potential confounding factors. However, low NLR with hyperglycemia and high NLR without hyperglycemia were not linked to any poor outcomes in this study. Furthermore, the interactive effect of the pattern of high NLR and presence of hyperglycemia on the 3‐month poor outcome was observed (P for interaction = 0.035). High NLR was merely a strong predictor of 3‐month poor outcome (adjusted OR (aOR) = 4.12; 95% CI, 1.57–10.75) and END (aOR = 4.30; 95% CI, 1.38–13.38; Figure 3) in stroke patients with hyperglycemia.

**TABLE 3:** Odds ratio and 95% confidence interval of NLR with hyperglycemia or not for outcomes at 3 months

Outcomes	*N* of cases (%)	Crude		Adjusted	
		Crude OR (95% CI)	*p* Value	Adjusted OR (95% CI)	*p* Value
Poor outcome at 3 months					
Non‐HR and non‐HG	33 (23.24)	1	—	1	—
HR and non‐HG	40 (43.01)	2.49 (1.42–4.39)	.002	1.27 (0.63–2.54)	.502
Non‐HR and HG	15 (28.30)	1.30 (0.64–2.66)	.466	1.04 (0.45–2.42)	.931
HR and HG	67 (72.04)	8.51 (4.68–15.47)	<.001	4.42 (2.13–9.16)	<.001
END					
Non‐HR and non‐HG	27 (19.01)	1	—	1	—
HR and non‐HG	28 (30.11)	1.89 (0.91–3.93)	.088	2.65 (1.16–6.07)	.210
Non‐HR and HG	13 (24.53)	1.61 (0.66–3.91)	.292	1.26 (0.45–3.49)	.656
HR and HG	35 (37.63)	3.75 (1.90–7.39)	<.001	4.81 (2.08–11.12)	<.001
Mortality at 3 months					
Non‐HR and non‐HG	6 (4.23)	1	—	1	—
HR and non‐HG	12 (12.9)	3.36 (1.21–9.29)	.020	1.88 (0.51–6.95)	.347
Non‐HR and HG	2 (3.77)	0.89 (0.17–4.55)	.888	1.15 (0.18–7.40)	.887
HR and HG	32 (34.41)	11.89 (4.73–29.92)	<.001	6.56 (1.92–22.40)	.003

Note

Adjusted for age, sex, cigarette smoking, alcohol consumption, systolic blood pressure, onset‐to‐treatment time, hypertension, hyperlipidemia, atrial fibrillation, previous stroke, baseline NIHSS score, triglyceride, total cholesterol, low‐density lipoprotein cholesterol, and high‐density lipoprotein cholesterol, HR high neutrophil‐to‐lymphocyte ratio, HG hyperglycemia.

Abbreviations: CI, confidence interval; OR, odds ratio.

## 4 DISCUSSION

The study investigated the association of NLR and blood glucose on prognosis of ischemic stroke patients treated with IVT. The major findings in this study included (a) patients with concurrent high NLR and hyperglycemia were associated with increased risks of END, 3‐month poor outcome, and 3‐month mortality, (b) high NLR and hyperglycemia exerted a significant multiplicative interaction on poor outcome at 3 months, and (c) high NLR was merely a strong predictor of 3‐month poor outcome and END in patients with hyperglycemia.

In recent decades, much more attentions have been paid to the prognostic values of hyperglycemia and inflammation in patients with ischemic stroke. Numerous studies showed hyperglycemia (Capes, Hunt, Malmberg, Pathak, & Gerstein, 2001; Masrur et al., 2015; Poppe et al., 2009) and inflammation (Gong et al., 2019; Kim et al., 2012; Maestrini et al., 2015) were linked to adverse outcomes in stroke patients with or without IVT treatment. In an analysis based on 1,098 ischemic stroke patients treated with rtPA, admission hyperglycemia was independently associated with increased risk of symptomatic intracerebral hemorrhage (SICH), death, and poor functional outcome at 3 months (Poppe et al., 2009). A meta­analysis also has shown that admission glucose level >6.1 mmol/L has an increased risk of 30‐day mortality in nondiabetic patients after stroke (Capes et al., 2001). On the other hand, neutrophils are generally the first and most abundant inflammatory cells to respond to ischemic stroke and migrate to cerebral ischemic regions during the whole acute phase of inflammation (Jin, Yang, & Li, 2010; Shichita, Sakaguchi, Suzuki, & Yoshimura, 2012). Prior studies found that neutrophil count was related to stroke severity at admission (Kim et al., 2012), SICH, and 3‐month mortality (Maestrini et al., 2015) in patients with stroke. Neutrophil‐to‐lymphocyte ratio (NLR) emerged as a systemic biomarker for inflammation. In terms of ischemic stroke, NLR has been identified as an independent predictor of unfavorable clinical outcomes (Qun et al., 2017). Jijun Shi at al. found that NLR was a dynamic variable and NLR at 24 hr after rtPA infusion was significantly associated with increased risks of death and major disability at 3 months (Shi et al., 2018). Experimental studies revealed that hyperglycemia enhanced neutrophil infiltration in the ischemic brain and exacerbated the brain lesion (Justicia et al., 2017; Lin, Ginsberg, Busto, & Li, 2000). In our study, elevated NLR was an independent risk indicator for poor outcome in stroke patients with hyperglycemia, while we did not observe any association between NLR and outcomes in patients without hyperglycemia. However, most of the previous studies did not offer such stratification. Our study found a significant interaction between NLR and blood glucose on 3‐month poor outcome in ischemic stroke patients after IVT. It has to be mentioned that NLR was not further stratified based on the levels of glucose in this study, which might underestimate the association between elevated NLR and poor outcome in patients without hyperglycemia.

The pathological mechanisms of adverse effect of hyperglycemia on outcomes after AIS are similar to the pathophysiological processes of inflammatory in experimental studies. The plausible explanations for this connection are stated as following. First, hyperglycemia is known to stimulate the proinflammatory transcription factors, such as nuclear factor κB (Esposito et al., 2002), increase the expression of intercellular adhesion molecule‐1 (ICAM‐1) and neutrophil infiltration (Justicia et al., 2017) and lead to ischemic tissue damage. Furthermore, Hyperglycemia could also increase inflammatory cytokine (including tumor necrosis factor‐α [TNF‐α], interleukin‐6 [IL‐6], and interleukin‐18 [IL‐18]) by an oxidative stress mechanism in human (Esposito et al., 2002), suggesting hyperglycemia triggers inflammatory response. Second, studies have demonstrated that hyperglycemia is associated with reperfusion injury (Martín et al., 2006; Tarr et al., 2013). Hyperglycemia is thought to enhance matrix metalloproteinase‐9 (MMP‐9) activity in the ischemic area during reperfusion, which reduces a junctional protein in endothelial cells and then results in blood–brain barrier (BBB) damage (Kamada, Yu, Nito, & Chan, 2007). It is worth noting that neutrophils have been shown to be an important source of MMP‐9 and may mediate HT through MMP‐9 in ischemic stroke (Gautier et al., 2014). As mentioned above, the pathological mechanisms of inflammatory responses and hyperglycemia on poor outcomes of AIS after IVT impact each other through several complex signal pathways, though the definite biological mechanisms are still insufficiently understood. Therefore, more large studies are needed to further investigate the precise mechanisms.

### 4.1 Study limitations

Our study has several limitations. First, the study population was relatively small from a single hospital, which inevitably produced selection bias and may limit the generalization of our findings. Second, NLR and blood glucose levels were only measured at around 24 hr after thrombolysis. Thus, without serial measurements, we have no data to examine the association between NLR and glucose change and stroke prognosis. Third, our registry had no record the use of hypoglycemic agents and what antiplatelet or anticoagulant medication they received after IV thrombolysis.

## 5 CONCLUSIONS

The combination of high NLR and hyperglycemia was a stronger predictor to 3‐month poor functional outcome, 3‐month mortality, and END in patients with acute ischemic stroke after IVT treatment. Based on this finding, strict control of inflammatory parameters and glucose may help to improve outcome for patients with ischemic stroke.

## CONFLICT OF INTEREST

None declared.

## AUTHOR CONTRIBUTIONS

Yani Cheng and Anna Ying conceptualized and designed the study, analyzed the data, and drafted the manuscript. Yanyan Lin and Junru Yu assisted in conceptualizing and designing the study. Ji Luo and Yifan Zeng collected data. YSL contributed to rewriting and editing the final version of the manuscript. All authors read and approved the manuscript.

### Peer Review

The peer review history for this article is available at https://publons.com/publon/10.1002/brb3.1741.

## Data Availability

The data that support the findings of this study are available from the corresponding author upon reasonable request.
